# New Photocrosslinked 3D Foamed Scaffolds Based on GelMA Copolymers: Potential Application in Bone Tissue Engineering

**DOI:** 10.3390/gels9050403

**Published:** 2023-05-11

**Authors:** Jesús L. Pablos, Javier Jiménez-Holguín, Sandra Sánchez Salcedo, Antonio J. Salinas, Teresa Corrales, María Vallet-Regí

**Affiliations:** 1 Departamento de Química en Ciencias Farmacéuticas, Facultad de Farmacia, Instituto de Investigación Sanitaria Hospital 12 de Octubre, imas12, Universidad Complutense de Madrid (UCM), 28040 Madrid, Spain; javiej03@ucm.es (J.J.-H.); salinas@ucm.es (A.J.S.); vallet@ucm.es (M.V.-R.); 2 Networking Research Center on Bioengineering, Biomaterials and Nanomedicine (CIBER-BBN), 28040 Madrid, Spain; 3 Grupo de Fotoquímica, Departamento de Química Macromolecular Aplicada, Instituto de Ciencia y Tecnología de Polímeros, C.S.I.C., Juan de la Cierva 3, 28006 Madrid, Spain; tcorrales@ictp.csic.es

**Keywords:** GelMa copolymers, tunable properties, cytocompatible polymers, cell proliferation, differentiation, tissue engineering, bone regeneration

## Abstract

The production of customized polymeric hydrogels in the form of 3D scaffolds with application in bone tissue engineering is currently a topic of great interest. Based on gelatin methacryloyl (GelMa) as one of the most popular used biomaterials, GelMa with two different methacryloylation degrees (DM) was obtained, to achieve crosslinked polymer networks by photoinitiated radical polymerization. In this work, we present the obtention of new 3D foamed scaffolds based on ternary copolymers of GelMa with vinylpyrrolidone (VP) and 2-hydroxyethylmethacrylate (HEMA). All biopolymers obtained in this work were characterized by infrared spectroscopy (FTIR) and thermogravimetric analysis (TGA), whose results confirm the presence of all copolymers in the crosslinked biomaterial. In addition, scanning electron microscopy (SEM) pictures were obtained verifying the presence of the porosity created by freeze-drying process. In addition, the variation in its swelling degree and its enzymatic degradation in vitro was analyzed as a function of the different copolymers obtained. This has allowed us to observe good control of the variation in these properties described above in a simple way by varying the composition of the different comonomers used. Finally, with these concepts in mind, biopolymers obtained were tested through assessment of several biological parameters such as cell viability and differentiation with MC3T3-E1 pre-osteoblastic cell line. Results obtained show that these biopolymers maintain good results in terms of cell viability and differentiation, along with tunable properties in terms of hydrophilic character, mechanical properties and enzymatic degradation.

## 1. Introduction

One of the most challenging topics currently is the design of biomaterials that may be recognized as scaffolds for cell culture in bone-tissue engineering applications. Those scaffolds should be able to mimic the biological and structural features of the extracellular matrix (ECM), as these modulate and direct cell behavior and in a manner that allows cells to proliferate and differentiate to regenerate the damaged tissue [1,2,3,4]. In particular, the search for a versatile matrix for bone tissue engineering to overcome difficulties such as insufficient mechanical properties or unrestrained degradation is of major relevance. There is a great need for new and innovative tools for the treatment of bone defects, whether they are caused by accidents or injuries. It is well known that natural bone tissue is a sophisticated material which is composed of inorganic and organic compounds with a complex structure. In relation to the bone remodeling process, the succession of five main steps must be highlighted: activation, resorption, reversion, formation and termination, in which coordination between several cell lineages at both a local and systemic level is crucial [5]. This process is mainly driven by intercellular communication and by the microenvironment in the affected region. In this sense, the attachment, proliferation, and differentiation of the cell lineages recruited are essential for the beginning of the bone remodeling process and will influence the final outcome.

Apart from the addition of inorganic components to biocompatible polymers to create biomaterials which allow mineralization and bone tissue regeneration [6,7], another widely used approach is the use of mixtures of different biodegradable organic materials (synthetic or natural polymers) to obtain hybrid hydrogels [8,9,10], so that their properties could be tuned by adjusting the ratio between them [11].

It should be noted that synthetic polymers will have better mechanical properties while natural polymers are preferred by cells. A widely used strategy is the application of mixtures of GelMa/organic biomaterial, such as polylactic acid, natural extracellular cartilage-derived matrix or organic-derived poly (ester amide) to form in all cases hybrid organic hydrogel scaffolds, which has proven to be an effective strategy for bone tissue regeneration [9,12,13,14,15].

When it comes to the hydrogel scaffold, we define it as three-dimensional, crosslinked networks of water-soluble polymers, that can swell in water without being dissolved and with a wide versatility of chemical compositions in terms of combinations of comonomers, their crosslinking density (in relation with methacryloylation degree, DM), the synthetic conditions and thus a wide range of tunable physical properties. Furthermore, they possess a porous structure, with many similarities to ECM, which favors an efficient exchange of nutrients, the adsorption of macromolecules involved in cell adhesion and allows vascularization of the scaffold [16]. Thus, hydrogels are broadly used in clinical practice and experimental medicine for a wide range of applications and above all in bone tissue engineering and regenerative medicine, from soft tissues (blood vessels) to hard tissues (bone cartilage), since this set of tunable properties have a positive effect on the cell adhesion, proliferation, migration and, subsequently, on the differentiation [17,18,19].

A key aspect in the field of bone tissue engineering is the choice of the set of biodegradable organic materials both synthetic and natural which will form the hydrogel and therefore the scaffold. At this point, the use of gelatin must be stressed, as one of the most widely studied natural hydrophilic polymers. This is extracted from the denaturation of collagen and exhibits many advantages, such as good cytocompatibility, solubility, low immunogenicity [20], the presence of cell adhesion motifs [21], good degradability and being easier to access. Because of these good properties, gelatin favors cell proliferation and differentiation, a key process in tissue engineering, since gelatin is considered a material with the possibility of emulating the natural structure of the ECM [22]. However, a major drawback is that its thermostability is poor; gelatin will only form a physical hydrogel at a concrete range of concentrations and temperatures, along with the potential toxicity of chemical crosslinking reagents. However, if we carefully examine the structure of gelatin, we can appreciate many active groups on side chains of gelatin (-OH, -COOH and -NH_2_) which open the possibility of modifying gelatin with specific groups. The most popular functionalization of gelatin is the introduction of methacrylamide groups. This arrangement allows a functionalized natural polymer (GelMa) to be obtained with the feature of photocrosslinking because of these methacryloyl groups, in an easy way under UV light along with a biocompatible photoinitiator. Thus, it is possible to obtain GelMa hydrogels covalently crosslinked, with outstanding thermostability and controlled porosity and stiffness by tuning the degree of functionalization, and, most importantly, maintaining the cytocompatibility and degradation properties of raw gelatin. This makes it widely used in biomedical applications [15,23].

Regarding other synthetic biodegradable organic comonomers to form mixtures, poly-(2-hydroxyethyl methacrylate) (pHEMA) is widely used, which is a chemically stable and biocompatible polymer and whose properties of permeability and hydrophilicity can be adjusted with use of different crosslinkers due to their lower water retention ability. However, it has a disadvantage, which is that pHEMA does not allow cell adhesion and proliferation on its surface which limits its biomedical applications when cell cohesion is required [24]. On the other hand, vinylpyrrolidone (VP) is known for its amorphous nature and for being one of the most used hydrophilic monomers for the synthesis of biomaterials with application in common biomedical and tissue engineering. Furthermore, it has good solubility in water and in some organic solvents and possesses inherent properties, such as stability at various pH, excellent thermal properties, easy crosslinking capacity and good mechanical strength which makes polyvinylpyrrolidone (PVP) an efficient biomaterial, even when used in pharmaceutical applications [25].

Bearing all these aspects in mind, among the wide range of hydrophilic polymers, the copolymers based on HEMA with VP are a promising candidate to be used as biomaterials [26,27] with the combination of the advantages of every monomer used in the new copolymer networks obtained. In addition, the synergistic effect of the presence of these monomers, VP or HEMA, is known in the biological properties of GelMa copolymers [25,28].

In this work, a collection of new ternary systems based on VP/HEMA copolymers (10 %wt. and 30 %wt.) has been prepared. In addition, GelMa homopolymers with higher and lower methacryloylation degree (H-GelMa and L-GelMa) as reference systems and copolymers with VP and HEMA (GelMa/VP and GelMa/HEMA) have been prepared to study the improvements provided by these new systems.

The main purpose of this work is to analyze the improvement of the physicochemical properties of the GelMa/VP/HEMA ternary systems proposed in this work to confirm a higher mechanical property, disentangle the possibility of obtaining a much slower and sustained enzymatic degradation profile and maintaining an adequate level of hydrophilicity similar to GelMa homopolymers. All of this occurs while maintaining equal or higher viability values and most importantly, by preserving the cell differentiation capacity with values close to H-GelMa and L-GelMa homopolymers as reference systems.

For this reason, in this work we propose the strategy of using ternary systems based on GelMa/VP/HEMA copolymers. It is important to note that, to the best of our knowledge, this is the first report in the obtention of these crosslinked ternary systems based on copolymers of GelMa/VP/HEMA, through radical photoinitiated polymerization.

## 2. Results and Discussion

In this work, we report on the synthesis together with the physicochemical and biological characterization of new crosslinked copolymers as porous scaffolds (in the form of foamed scaffolds obtained by freeze-drying process). These new biopolymers are based on the synthesis of the gelatin methacrylated monomer with two methacryloylation degrees (higher DM and lower DM), copolymerized with vinylpyrrolidone (VP) and/or 2-hydroxyethylmethacrylate (HEMA) comonomers. In this way, two different sets of materials have been obtained, as binary and ternary systems for every methacryloylation degree (Table 1).

This study is based on the strategy of obtaining polymer systems with improved properties as the biomaterial when compared to the properties of the single components, using two well-known polymers used in biomedical applications, such as PVP and pHEMA [24,25,29]. The obtained results described below show that with this strategy we can easily obtain different materials, with a good degree of swelling, different properties in relation to their in vitro enzymatic degradability and easily modulable depending on the application. The use of systems based on crosslinked materials of GelMa, and VP/HEMA show an improvement in their biological response against pre-osteoblastic cell lines and their differentiation to osteoblastic cells, which open a promising strategy in scaffolds’ obtention and their application in tissue engineering and bone regeneration. The main purpose of this work lies in the fact that the presence of the different comonomer mixtures translates into a synergy between them that favors an improvement in its cytocompatibility while improving their physico–chemical properties, in terms of a tunable swelling degree and in vitro enzymatic degradation.

### 2.1. Synthesis of GelMa with Two Methacryloylation Degrees (DM)

Gelatin methacrylamide (GelMa) is one of the most widely used type of photopolymerizable gelatin-based derivatives in biomedical applications, due to its ability to easily form hydrogel structures through radical photopolymerization [29]. GelMa is obtained from gelatin, which is the product of the hydrolysis and denaturation of collagen. Their synthesis process is based on the reaction of the free amino groups of lysine and hydroxyl lysine residues in gelatin with methacrylic anhydride in phosphate-buffered saline (pH = 7.4) obtaining degrees of substitution between 70% and 85% [30].

A method of obtaining higher methacrylation degrees and to modulate the degree of methacrylation with a lower anhydride ratio is described by Shirahama et al. [31]. The use of a carbonate–bicarbonate buffer allows the simplification of the synthesis process by reducing the ratio of methacrylic anhydride used and modulating the degree of methacrylation in a more efficient way.

**Figure 1:**
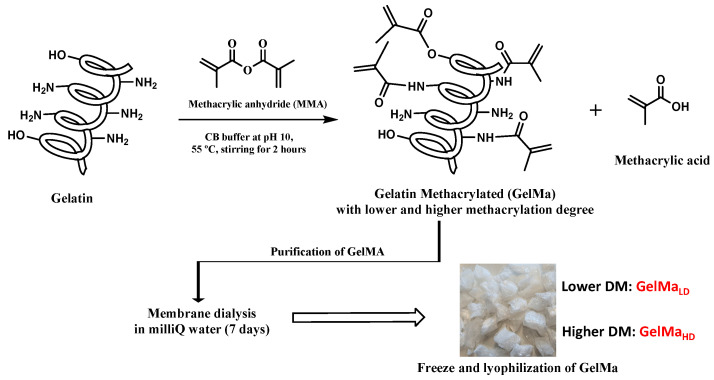
Scheme synthesis of GelMa monomers with two methacryloylation degrees and subsequent purification by reacting with methacrylic anhydride at basic pH. For GelMa purification, a dialysis tubing cellulose membrane (with 14 KDa molecular weight cut-off) was used, followed by its subsequent freeze and lyophilization.

In this work, gelatin type A (Isoelectric point around pH 9) was used. As described in the Materials and Methods section, the reaction was achieved by adding methacrylic anhydride (MA) in two ratios (0.2 mL/g gelatin and 0.05 mL/g gelatin), dropwise into a gelatin solution in the carbonate buffer (0.2 M, pH 10) under vigorous stirring, following by the removal of residual MA as well as methacrylic acids by dialysis for 7 days to finally obtain the foam-like GelMa after freeze-drying (Figure 1).

**Figure 2:**
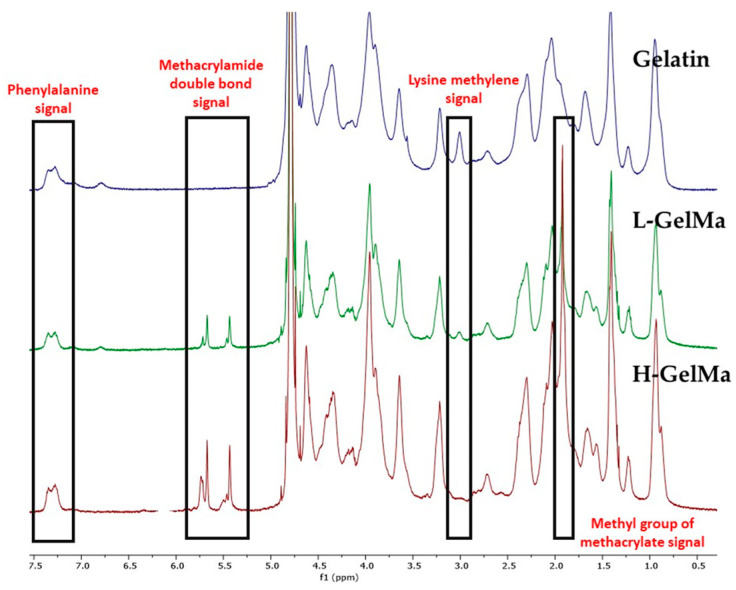
^1^H NMR spectra of unmodified gelatin and methacrylated gelatin with two different methacrylation degrees (H-GelMa and L-GelMa). The signals of the acrylic protons and the methyl function of the introduced methacrylic groups are indicated (1.8–1.9 ppm and 5.3 ppm/6.2 ppm) together with the lysine methylene signals of gelatin at 2.9 ppm. DM was calculated following Equation (1): DM(%) = (1 − Area_lys meth GelMa_/Area_lys meth Gelatin_) × 100.

The successful functionalization of gelatin was verified by ^1^H-RMN spectroscopy (Figure 2). The appearance of new signals in functionalized gelatin can be observed. The following signals must be highlighted: Peaks at 5.3 ppm and 6.2 ppm together with the signal at 1.8–1.9 ppm, which are assigned to the acrylic protons and the methyl group of the introduced methacrylic groups, respectively. In general, these peaks were higher in the higher DM samples.

A quantitative analysis of the different ^1^H-RMN spectra obtained was carried out to determine the extent of the functionalization of free amino groups into methacrylamide groups (DM). These values were obtained by comparing the signals of unmodified gelatin by using as a reference the peak area of aromatic acids (phenylalanine signal at 7.3 ppm) in the gelatin and GelMa samples in each spectrum (peak at 7.4 ppm). Then, by comparing the peak area of lysine methylene protons which appear at around 2.9 ppm (Equation (1)) it was possible to determine the DM of every GelMa synthesized (H-GelMa and L-GelMa).

Thus, the following values of DM were achieved; for the highest DM, the value obtained was 92% and the lowest value was 70.5%.

These results indicate the possibility of obtaining a control on the degree of methacrylation and therefore on the crosslinking density, which led to biopolymers with tunable properties. In addition, it must be noted that a higher DM will result in a higher storage modulus, as described by Lee, B. et al. [32]

### 2.2. Biomaterial Preparation and Characterization GelMa and GelMa Copolymers

#### 2.2.1. Biopolymer Preparation

- Why this strategy?

GelMa is considered as a versatile polymerizable material that possesses the ability to be incorporated with other polymerizable materials, such as PEGDMA, pHEMA, PVP and a wide variety of biocompatible polymers [18,25,33,34,35] to create tunable GelMa-based copolymers in the easy way through radical photopolymerization. The main purpose is to take advantage of the presence of those copolymers in biopolymer structure, in terms of improving their biological properties.

With this objective in mind, the strategy followed in this work has been the use of water-soluble monomers that result in materials with excellent chemical, thermal and biological stability, such as N-vinylpyrrolidone (VP) and 2-hydroxyethylmethacrylate (HEMA).

For one side, pHEMA, chemically stable and biocompatible polymer and with adjustable properties [24]. On the other side, VP, one of the most used hydrophilic monomers for the synthesis of biomaterials with application in common biomedical and tissue engineering [25]. With these two items in view, we have developed an in-depth study based on copolymers of HEMA with VP as a promising candidate to be used as biomaterials [26,27] with the combination of the advantages of every monomer, and the analysis of synergistic effect of their presence in biological properties of GelMa copolymers [25,28]

**Figure 3:**
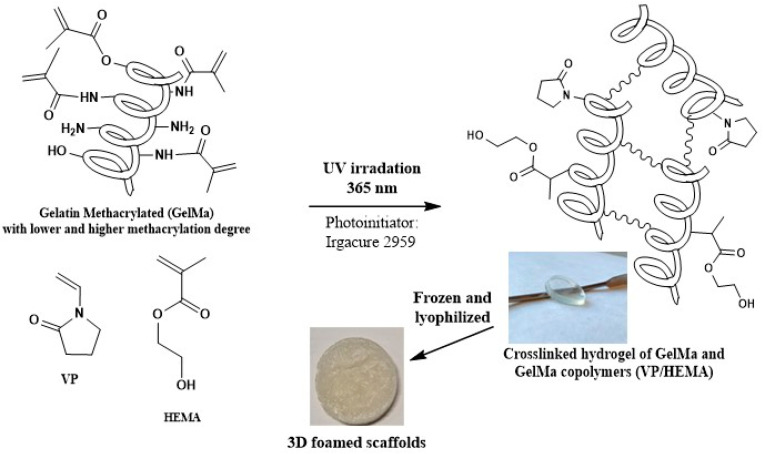
Scheme of synthesis of biopolymers synthesized: Homogeneous solutions of GelMa and/or comonomers (VP and HEMA) to obtain by UV radical photopolymerization the crosslinked homopolymers and/or copolymers with vinylpyrrolidone and/or HEMA. It is also possible to observe the digital pictures of materials before and after lyophilization.

For this reason, copolymers of GelMa with HEMA/VP comonomers were obtained, with two different concentrations (10 %wt. and 30 %wt.) of 1:1 mixtures of HEMA/VP, together with those compositions in GelMa/VP and GelMa/HEMA copolymers (Figure 3, Table 1).

- Biopolymer synthesis

As described in the Materials and Methods section, all materials used in this work were prepared the easy way by bulk radical photopolymerization using water as the solvent. After dissolution of all comonomers used in each composition, mixtures were degasified by using ultrasounds and obtaining in all cases homogeneous solutions of GelMa and the corresponding comonomers together with the photo-initiator. Finally, by placing the mixtures in circular Teflon molds, after 20 min of UV irradiation, it was possible to obtain the different crosslinked biomaterials whose constitution is depicted in Figure 3 and Table 1. To induce the internal porosity of biomaterials, these are freeze-dried.

In this way, it was possible to obtain a collection of different freeze-dried materials, with porous structure and good mechanical properties in terms of manageability, as can be seen in Figure 3.

#### 2.2.2. Biopolymer Characterization

The materials obtained in this work were characterized in terms of disentangling their physico-chemical properties together with the variation in these as a function of the composition of the different biopolymers synthesized and that will influence its behavior as a biomaterial.

- FTIR spectroscopy

**Figure 4:**
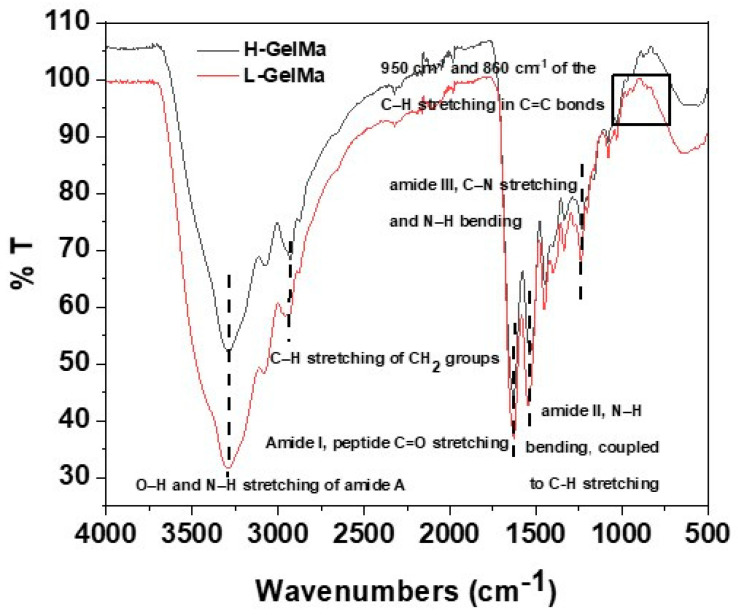
FTIR spectra of GelMa monomer with two methacryloylation degrees (L-GelMa and H-GelMa samples). Inside are described the most relevant bands related to the GelMa monomer.

All biopolymers along with GelMa monomers (H-GelMa and L-GelMa) were characterized by FTIR (Thermo Scientific™, Waltham, MA, USA). Firstly, spectra of GelMa monomers were obtained. As can be observed in Figure 4, results obtained reveal the characteristic absorption bands present in the backbone structure of gelatin. Signals can be observed around 3320 cm^1^ (O–H and N–H stretching of amide A), 2930 cm^1^ (C–H stretching of CH_2_ groups), 1645 cm^1^ (amide I, peptide C=O stretching), 1540 cm^1^ (amide II, N–H bending coupled to C–H stretching) and 1240 cm^1^ (amide III, C–N stretching and N–H bending). In general, it must be noted that there are not significant changes in GelMa monomers regarding the unmodified gelatin [36] because the contribution of the methacrylic double bound (C=C) stretching appears in the range of 1680–1650 cm^1^. However, there is evidence of the presence of methacrylate groups in the gelatin structure; GelMa samples show two bands around 950 cm^−1^ and 860 cm^−1^ which are assigned to the C–H stretching of C=C. These results, in conjunction with ^1^H-RMN evidence, reveal that methacrylate groups have been introduced into the structure of the gelatin.

**Figure 5:**
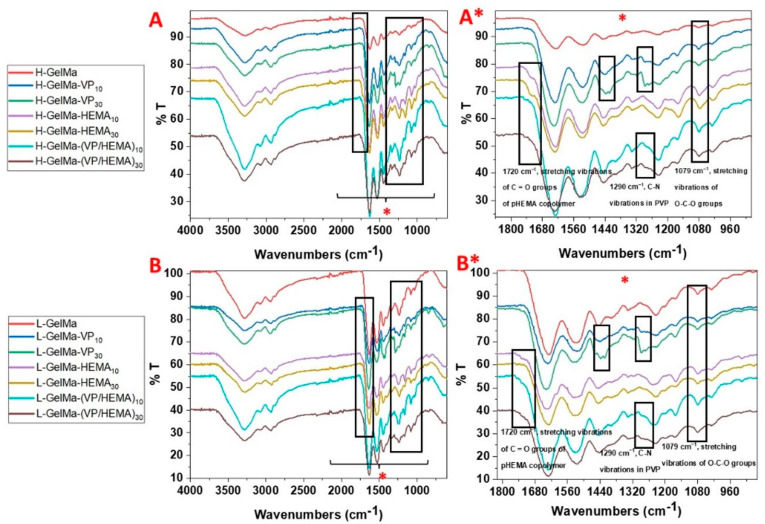
(**A**,**B**) FTIR spectra of H-GelMa and L-GelMa biopolymer families copolymerized with vinylpyrrolidone (VP_10/30_), HEMA (HEMA_10/30_) and vinylpyrrolidone/HEMA (VP/HEMA_10/30_). (A*,B*) Expansion of the region between 1800 and 950 cm^−1^. Inside are described the most relevant bands related to the each comonomer present in every composition proposed.

Moreover, the FTIR of all biopolymers synthesized was obtained to confirm the presence of different comonomers used in the compositions as is detailed in Figure 5. Therefore, the FTIR spectra were analyzed to point out the characteristic bands of each of the different comonomers used, VP and HEMA. First, in copolymers of GelMa with VP, the presence of the bands of GelMa described in Figure 4 can be observed, together with the evidence of the characteristic signals of PVP: deformational vibrations of CH- group in PVP at 1320 cm^−1^ and signals at 1415 cm^−1^ and 1480 cm^−1^ [2]. This result confirms the presence of PVP in copolymer synthesized.

On the other hand, copolymers with HEMA show characteristic bands around 1079 cm^−1^ which are related to the stretching O–C–O groups and a shoulder at around 1720 cm^−1^ that corresponds to the stretching vibrations of carbonyl groups [37], which confirms their presence in the copolymer. The higher intensity of the bands described above for materials with a higher content of the different copolymers used (10 %wt. and 30 %wt.) must also be highlighted.

Finally, if we analyze the spectra of the ternary systems based on GelMa/VP/HEMA, the bands related to both comonomers can be found again, which confirms the proposed structure. In addition, a higher intensity of the bands is again observed in the systems with the higher comonomer content.

The results achieved confirm the correct obtaining of the biomaterials proposed in this work.

- Thermal properties: TGA analysis

Thermal degradation of homopolymers H-GelMa/L-GelMa and copolymers with VP and/or HEMA were studied by determining the weight loss of each sample using a temperature ramp from 25 °C to 800 °C at 10 °C/min, in nitrogen atmosphere by conventional thermogravimetric analysis (TGA). Table 2 sets out the thermogravimetric data for all biopolymers in relation to the temperature at 10 %wt. weight loss (T_10_) and the maximum decomposition temperature (T_max_), which was collected from the derivative weight loss curve (DTGA).

It can be observed in thermograms for GelMa homopolymers that T_max_ occurred at around 318 °C, as is depicted in Table 3. Data obtained in thermograms for copolymers confirm the presence of these which translate into the appearance of a new peak of T_max_ in all copolymers. It is shown around 430 °C for VP copolymers [38], 415 °C for HEMA copolymers [39] and between them for copolymers of VP/HEMA. The presence of those comonomers improves the thermal stability of GelMa homopolymers and enhances the values of T_10_.

- Swelling behavior

One key parameter in biomaterials is the water-sorption capacity. A high swelling degree behavior makes them excellent candidates as highly cytocompatible materials. It must be highlighted that one of the requirements of these types of materials is their high hydrophilicity, resulting in weak interactions with the extracellular matrix components [17]. On the other hand, it is well known that higher methacryloylation degrees led to a rise in the mechanical strength and a reduction in the swelling degree of the resulting gelatin hydrogels [1].

Bearing these aspects in mind, in this work we skillfully chose the methacrylation degree, both higher and lower, to modulate the swelling behavior of biopolymers obtained. Moreover, we investigated the impact of the copolymerization of chosen comonomers (VP and/or HEMA) in their swelling behavior by using different media, such as milliQ water, physiological medium (PBS) and culture cell medium (α-MEM).

**Figure 6:**
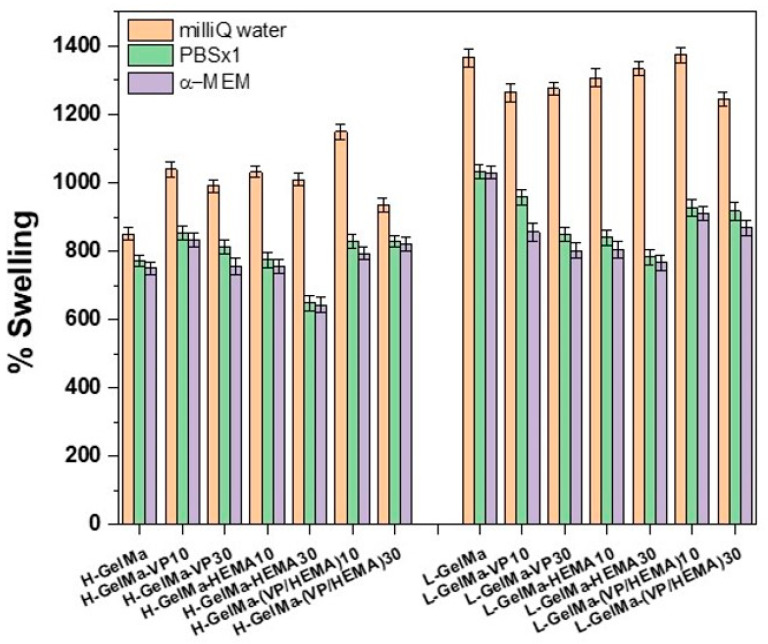
Degree of swelling of the different biopolymers after 24 h in different media: MiliQ water, PBS×1 and α-MEM. Data were calculated from Equation (2). Data were presented as mean ± standard deviation (SD). Error bars represented the SD of 3 independent samples. Statistical analysis was performed with the nonparametric Kruskal–Wallis test and post-hoc Dunn’s test. For all statistical tests, a value of *p* < 0.05 was considered significant.

As is depicted in Figure 6, we can vary the degree of swelling from around 800% for higher methacrylated gelatin material to around 1400% in the lower methacrylated gelatin material, which agrees with other authors [1]. These variances observed are caused by different reasons. Firstly, it must be highlighted that the water-binding capacity of proteins depends on various parameters, such as, for example, steric factors or amino acid composition of protein or size; in this case, if we consider the polar amino groups, which are the primary sites of interaction between protein and solvent, the degree of methacryloylation leads to the elimination of this charged amino group and thus a lower swelling degree, as can be observed in Figure 6 by comparing both of the methacryloylation degrees, which range from 850% to 1450% for H-GelMa and L-GelMa, respectively.

In a similar way, we also succeeded in varying this parameter with the preparation of PVP and/or HEMA copolymers. For H-GelMa copolymers, no major improvements in swelling degree values were noted due to the higher degree of crosslinking of the biomaterial. On the other hand, for lower methacrylated materials, a slight decrease in swelling degree values can be noted for all copolymers obtained but maintaining sufficiently high values for their application as cytocompatible materials. This could be explained by the fact that the copolymerization of VP and/or HEMA monomers may result in larger mesh sizes and, therefore, in a lower capability to take up water due to a more crosslinked structure.

In general, it can be said that the swelling degree values remain generally high even in the cases of copolymers with VP and/or HEMA.

- Determination of porosity by SEM

**Figure 7:**
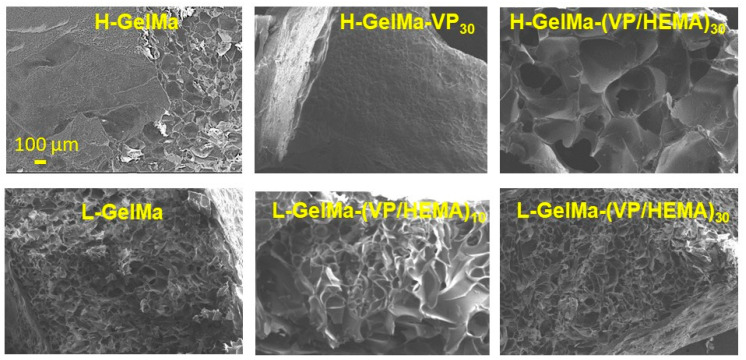
SEM micrographs of foamed scaffolds (L-GelMa/H-GelMa and copolymers). Images have been obtained from their cross-sectional view.

Porosity is another important parameter in the optimization of porous artificial bone grafts that can promote bone regeneration by its action as provisional templates for vascularized bone growth. Some key features of scaffolds are porosity and high interconnectivity, which are necessary to enable tissue growth, through osteogenesis, cell migration and nutrient transport [25,40]. Additionally, the size of the osteoblast is around 10–50 µm although they prefer larger pores of at least 100 or 200 µm. For this reason, the strategy followed in this work to create a porosity of at least 100–200 µm was to freeze-dry the materials obtained. To confirm the presence of this porosity, SEM analysis was performed through cross-sectional pictures of biomaterials obtained. The SEM images of some of the synthesized biopolymers are depicted in Figure 7.

It should be noted a homogeneous porosity around 100–300 μm can be observed in all the materials. If we analyzed in depth all SEM pictures obtained, a higher porosity can be observed in more crosslinked biomaterials, which agrees with results obtained by other authors [41] In any case, the main result is the confirmation of an adequately sized porosity that will facilitate the cell adhesion and subsequent differentiation of pre-osteoblastic cells. It is important to highlight the possibility of modulating the porosity obtained depending on the degree of crosslinking of the material, which depends on the DM obtained in the GelMa synthesis.

- In vitro enzymatic degradation

Another parameter of major relevance is the analysis of the in vitro degradation of the porous scaffolds. This is a key setting in the design of biomaterials with application in tissue engineering since the degradation behavior will determine their in vivo properties in terms of the rate of degradation of the biopolymer and their stability in a living body together with the release of bioactive species.

The ideal situation is that this degradation rate should not be higher than the regeneration rate, and likewise, should not be lower than the rate of regeneration of the damaged tissue, so that tissue healing, and the degradation rate should be synchronized. In this way, the material can provide optimal structural support until the corresponding family cells are able to generate the corresponding repaired tissue [42].

**Figure 8:**
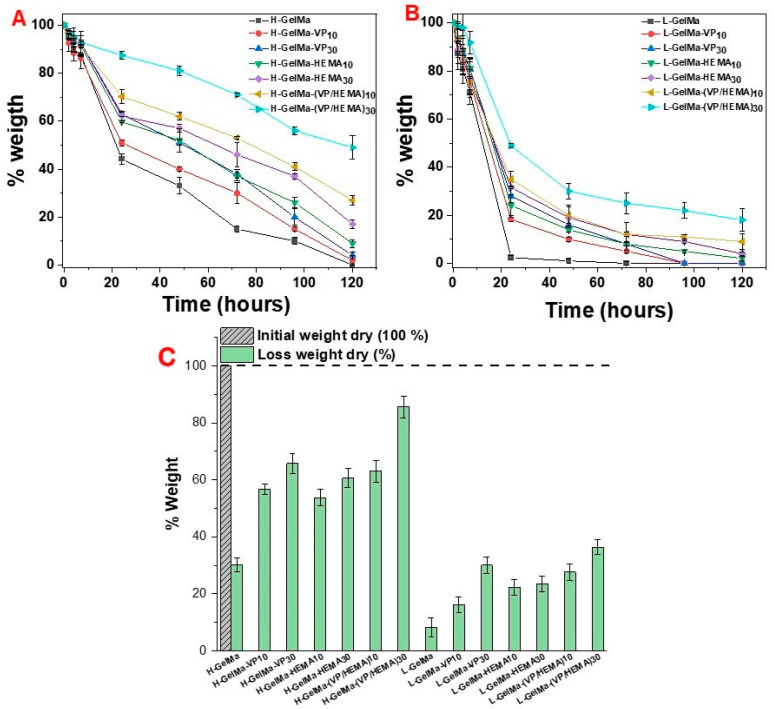
Enzymatic degradation of all different biopolymers synthesized. Collagenase type I degradation in all biopolymers synthesized in this work was studied at 2 h, 4 h, 7 h, 24 h, 48 h, 72 h, 96 h and 120 h. Results were analyzed in terms of their weight loss expressed as % weight loss, along with the weight loss of the dry initial sample and after 24 h of collagenase treatment. From left to right, (**A**). H-GelMa family and (**B**). L-GelMa family using a dilution of collagenase I (0.3 mg/mL) in DMEM for up to 24 h. (**C**). Determination of the dry weight of the biopolymers at zero time (100%) and after 24 h in terms of the remaining weight (%). Data were presented as mean ± standard deviation (SD). Error bars represented the SD of 3 independent samples.

In Figure 8, the results can be observed for collagenase type I degradation in all biopolymers synthesized in this work at 2 h, 4 h, 7 h, 24 h, 48 h, 72 h, 96 h and 120 h by analyzing the weight loss, along with the weight loss of the dry initial sample and after 24 h of collagenase treatment.

Significant differences in the degradation behavior of biopolymers depending on the crosslinking degree and different copolymer compositions are observed.

First, it must be highlighted that the degree of methacryloylation plays a key role in the degradation rate of these materials. At 24 h, the L-GelMa scaffold degradation is practically 100%, whereas for H-GelMa scaffolds, it is only about 45%, on account of the higher DM, which implies a more crosslinked material. It should be noted that after 5 days the degradation of H-GelMa is complete.

With respect to copolymers, the presence of comonomers generates a lower degradation degree of the system in both families of materials, increasing the resistance of those families to enzymatic degradation, ranging from slightly slower degradation rates in VP and HEMA copolymers with 10 %wt. to a lowest degradation rate observed in VP/HEMA 30 %wt. material, for which the degradation is around 50% in five days, since pHEMA is not degradable by collagenase [6].

The obtained results confirm the possibility of modulating the physico–chemical properties of biopolymers according to its comonomer composition in an easy way, obtaining a good variability on the degradation rates depending on the type of comonomer used, from a faster degradation if we use a GelMa with a lower degree of methacrylation to a more sustained degradation in time for the one with a higher degree of methacrylation. In addition, it is possible to modulate the degradation rate over time using VP/HEMA copolymers and varying their composition as shown in Figure 8.

- Rheological characterization

**Figure 9:**
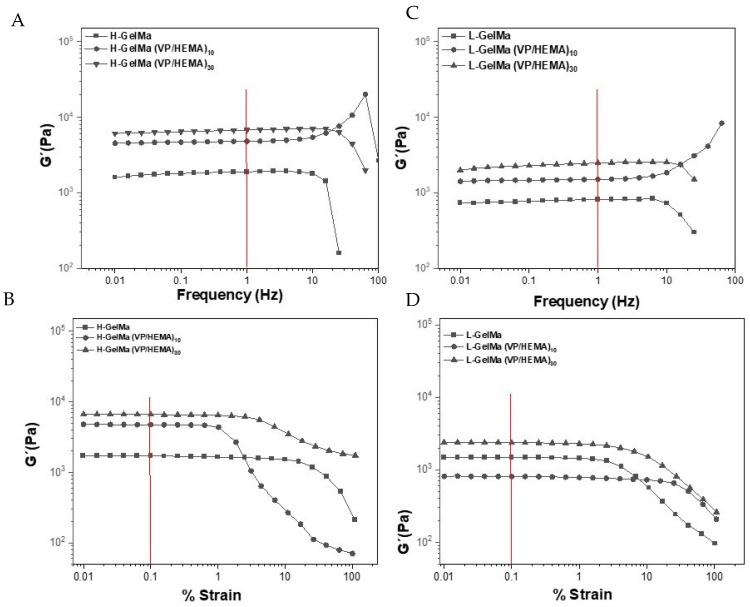
Rheological characterization of biomaterials in terms of storage modulus values (G′). (**A**,**C**) Frequency sweep sequence of biomaterials based on H-GelMa and L-GelMa, respectively. (**B**,**D**) Strain sweep sequence of biomaterials based on H-GelMa and. L-GelMa, respectively.

To analyze the stiffness of the proposed new copolymers and the influence of the presence of the VP/HEMA copolymers, the rheological properties of swollen GelMa-based biomaterials, H-GelMa and L-GelMa along with the ternary systems with VP/HEMA (10% and 30%) were studied. As can be observed in Figure 9, these biomaterials behave as glassy solids, with a typical frequency response for a gel-like material with a covalent polymer network formed by crosslinking polymer chains [43,44]. Table 3 shows the values of G′ obtained in frequency sweep and strain sweep tests. These values allow us to obtain a forecasting of the strain energy that is reversibly stored in a material, and which is characteristic of the elastic behavior of the material. The values of G′ were obtained at 1 Hz and 0.1% strain, respectively, where the values remain constant.

**Table 3:** Values of G′ obtained from frequency sweep and strain sweep tests, at 1 Hz and 0.1% strain, respectively, (linear viscoelastic region, red line in Figure 9) for homopolymers H-GelMa and L-GelMa and ternary systems based on VP/HEMA copolymers.

Higher DM			Lower DM		
Acronym	G′ (Pa, at 1 Hz)	G′ (Pa, at 0.1% Strain)	Acronym	G′ (Pa, at 1 Hz)	G′ (Pa, at 0.1% Strain)
H-GelMa	1895	1801	L-GelMa	813	805
H-GelMa-(VP/HEMA)_10_	4796	4729	L-GelMa-(VP/HEMA)_10_	1500	1470
H-GelMa-(VP/HEMA)_30_	6770	6543	L-GelMa-(VP/HEMA)_30_	2428	2359

Firstly, it can be noted that the softest materials are the homopolymers, H-GelMa and L-GelMa, with values of G′ around 1895 Pa and 813 Pa, respectively. These values agree with a material with a higher crosslinking degree, which involves a more rigid system. On the other hand, it must be highlighted that the copolymers based on VP/HEMA are more rigid, presenting a higher storage modulus value which increases with the higher copolymer content in the biomaterial. Although the content of water could affect the rheological properties of biomaterials [24], a drastic increase in storage modulus can be observed for H-GelMa (VP/HEMA)_30_ and in general for ternary systems proposed, being more pronounced with the increasing degree of crosslinking. This behavior can be attributed to the modification in the mobility of the polymer chains due to the presence and increasing amount of synthetic copolymers. These results are significant regarding their applications since it confirms that the variation in the composition affects the mechanical properties of the resulting tunable materials, which in turn connects with their influence in the degradation profile of the biomaterials.

### 2.3. Cell Response to Biopolymers

The MC3T3-E1 cell type is a pre-osteoblastic cell line derived from murine calvaria that presents a series of characteristics that are attractive for its use as an in vitro model, presenting values of proliferation, mineralization, and ALP activity in front of ascorbic acid and β-glycerol phosphate, such as primary human osteoblasts. For this reason, it is used as an archetypal model of osteoblast development in vitro [45]. Various biological parameters such as cell viability, proliferation, and differentiation for MC3T3-E1 pre-osteoblasts cells were addressed to evaluate their cell response to the different biopolymers studied, targeted at bone tissue engineering.

#### 2.3.1. Cell Viability and Proliferation of MC3T3-E1 Cells Exposed to GelMa-Based Biopolymers

**Figure 10:**
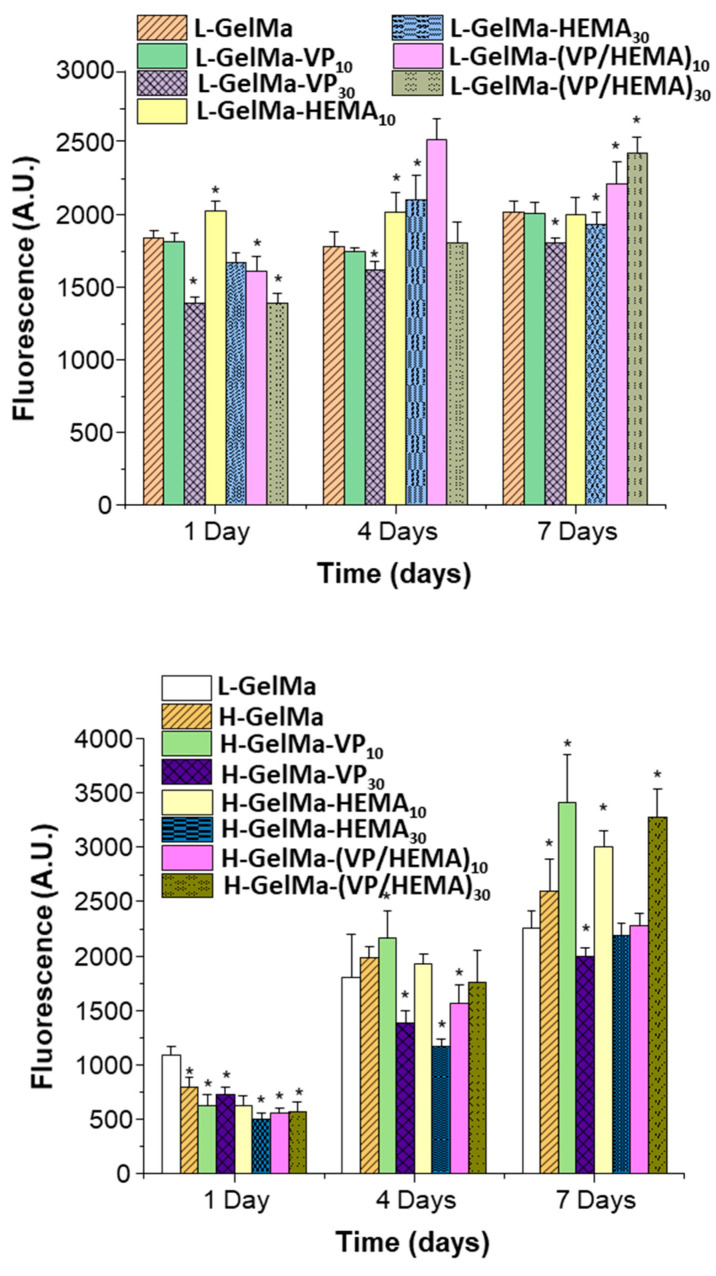
Proliferation of MC3T3-E1 pre-osteblast-like cells as a function of culture time onto H-GelMa and L-GelMa scaffold families, after 1, 4 and 7 days. * Comparisons between each H-GelMa and L-GelMa with every series. Data are presented as mean ± standard deviation (SD). Error bars represented the SD of 3 independent samples. Statistical significance: * *p* < 0.05 vs. H-GelMa or L-GelMa at 1, 4 and 7 days of culture.

Viability assays on the H-GelMa and L-GelMa scaffold families were carried out on MC3T3-E1 pre-osteoblastic cells at 1, 4 and 7 days and the results obtained are shown in Figure 10. Firstly, it should be noted that in the case of both families of biopolymers, H-GelMa and L-GelMa, the copolymers obtained in general present high cell viability.

The results obtained are consistent with the studies that were reported on the design of GelMa copolymers with VP or HEMA. In the case of the gelatin-PVP biomimetic polymer composite scaffold, they indicate biocompatibility and osteoinductive potential [25] together with the results obtained for the copolymers with HEMA which possess significant potential for use in corneal stromal mimics [28]. In the case of the L-GelMa-based biomaterials, it must be highlighted that a higher cell viability can be seen in L-GelMa-HEMA_10_, L-GelMa-HEMA_30_ which agrees with the results obtained for other authors, due to the synergy between GelMa and HEMA [33,46] However, the relevant result is that obtained for the copolymer composed of VP and HEMA, L-GelMa-VP/HEMA_10/30_. It should be emphasized that it is significantly better than their L-GelMa control, which indicates that there is an even more effective synergy in the same way as when using a ternary system but varying its physicochemical properties as described above. In addition, for the case of the H-GelMa family of copolymers, the compositions with the best cellular response are H-GelMa-VP_10_, H-GelMa-HEMA_10_ and once again, we find the best results for the ternary system H-GelMa-(VP/HEMA)_30_. In summary, it must be highlighted that the results obtained show a high viability and therefore proliferation on the surface of these materials for both families, even exceeding the values obtained by their respective controls at short times.

The best results are relative to the ternary systems proposed, which is the most relevant result, since it is necessary to obtain a fast enough cellular response to overcome the rate of degradation of the biomaterials because of enzymatic activity; thus, the degradation of the scaffolds is coupled with the formation of bone tissue at the same rate. These results indicate that GelMa copolymers with VP/HEMA present a more attractive surface for pre-osteoblasts than the GelMa-based polymer alone. This opens up the possibility, by modifying their physicochemical properties in terms of swelling degree and enzymatic degradation behavior, of maintaining a good cell viability response depending on the application requirements.

Furthermore, it must be stressed that in the materials with a higher degree of methacrylation cell proliferation is slower especially at day 1, however at longer times it reaches a higher cell viability of up to 30%. Considering that all the compositions are cytocompatible, it is possible to deduce that the variables that most affect pre-osteoblast development would be on one side, the degree of methacrylation of the GelMa and on the other, the incorporation of copolymers such as VP and/or HEMA, which contributes very positively to the cell development of the pre-osteoblasts while modifying their properties as described above.

However, at a lower degree of methacrylation, the presence of copolymers is not as decisive as the degree of methacrylation. As observed in the results described in Figure 8 in relation to the “in vitro” enzymatic degradation assay for all different biopolymers, it can be observed that approximately more than 50% of mass is lost in the L-GelMa family in comparison with H-GelMa family. These results could indicate that even though this family of materials presents high cytocompatibility values at short times, if it presents a faster degradation rate, it could end up compromising the stability of the scaffold and, therefore, reduce the surface of materials available to the osteoblasts to synthesize a new bone matrix.

#### 2.3.2. Cell Differentiation of MC3T3-E1 Cells Exposed to GelMa Based Copolymers

Osteoprogenitor cells are located on the endosteal and periosteal surface of the bone and their differentiation process is a crucial stage by which premature osteoblasts (pre-osteoblasts) must be transformed into mature osteoblasts, a process that ends with mineralized nodule formation [8]. It is a key parameter to obtain materials that meet the requirements of good compatibility and hosting properties with the aim of promoting bone cell growth.

In this way, it is essential that the material can be colonized by pre-osteoblast cells and that these can differentiate into osteoblasts, which ultimately will be responsible for forming the bone extracellular matrix and, therefore, for repairing the bone defect.

**Figure 11:**
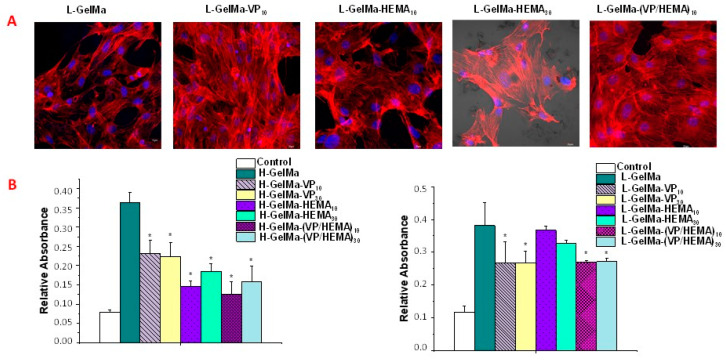
(**A**). Confocal laser scanning microscopy images of pre-osteoblast cultured onto scaffolds of L-GelMa family. (**B**). Histograms corresponding to the mineralization nodules of MC3T3-E1 stained with Alizarin-Red after being in contact with the biopolymers after 10 days of culture, respectively, on H-GelMa and L-GelMa families of biopolymers. Data are presented as mean ± standard deviation (SD). Error bars represented the SD of 3 independent samples. Statistical significance: * *p* < 0.05 vs. H-GelMa or L-GelMa after 10 days of culture.

With respect to the interaction between biomaterials and pre-osteoblast cells, 3D confocal microscopy studies were carried out on the H-GelMa and L-GelMa families of biopolymers scaffolds studied after MC3T3-E1 seeding (Figure 11A). To determine the cell morphology after 10 d., the surface of foamed scaffolds was analyzed. For it, Atto 565-phalloidin was used as the fluorescence probe of F-actin microfilaments. Furthermore, for nucleus die, DAPI fluorostaining was used. Proof of the fact that every type of biopolymer tested is cytocompatible, was found in the confocal laser scanning microscopy images of preosteoblasts cultured onto scaffolds, which present their typical spindle-shaped morphology, together with an adequate spread on the surface.

An indicator that reflects osteogenesis is the ability of mineral deposition and is considered as a marker of bone regeneration. Figure 11B shows the extracellular matrix mineralization nodules used as a late marker of the osteoblast differentiation process of MC3T3-E1 on the biopolymer scaffolds stained with Alizarin-Red. In both copolymer families, a significantly higher accumulation of calcium deposits is observed than in the pre-osteoblast-only control. Although, in the case of both the H-GelMa and L-GelMa family the presence of VP and/or HEMA does not improve pre-osteoblast cell differentiation with respect to H-GelMa or L-GelMa alone; therefore, we can state that in the case of the different copolymers obtained from VP/HEMA, there is no impediment to cell differentiation since they facilitate cell adhesion and cell viability as observed in the results described above and therefore, bone regeneration.

## 3. Conclusions

New biopolymers based on GelMa copolymers with VP/HEMA as comonomers were obtained for the first time as a proof of concept of the cytocompatibility of this type of new materials. For this, in the first place, GelMa with two DMs were synthesized and characterized. Secondly, the different biomaterials described above were obtained as homogeneous materials in a simple way by radical photopolymerization, in the form of homopolymers as reference materials (H-GelMa and L-GelMa) and copolymers with VP, HEMA and VP/HEMA (new proposed ternary systems).

Secondly, all copolymers obtained were characterized by infrared spectroscopy (FTIR) and assessing their thermal properties, confirming the presence of the different copolymer in each synthesized composition. The modification of those decisive parameters was studied which are implied in their cytocompatibility, such as the hydrophilicity of the materials in terms of a good swelling degree and a tunable enzymatic degradation. In addition, the mechanical properties of these ternary systems were studied in comparison with H-GelMa and L-GelMa homopolymers.

It can be seen how the ternary systems of H-GelMa/L-GelMA copolymers have a clear improvement in their mechanical properties, in terms of an increase in the storage modulus value (G′), this being more pronounced in H-GelMa ternary copolymers (from G′ values of 1895 Pa in H-GelMa to 6770 Pa in H-GelMa ternary systems). Furthermore, the possibility of obtaining a much slower and sustained enzymatic degradation profile in these ternary copolymer systems was observed, being the slowest in the case of ternary systems with H-GelMa (40% weight loss in 5 days compared to 100% for homopolymers). All of this occurred while maintaining a similar level of hydrophilicity regarding GelMa homopolymers with values around 800–1000% in all biomaterials obtained, together with equal or higher viability values and most importantly, maintaining the differentiation capacity with values close to H-GelMa and L-GelMa homopolymers. It is important to highlight that the two families L-GelMa and H-GelMa together with their corresponding copolymer families with VP and/or HEMA are cytocompatible against pre-osteoblastic cells, allowing its differentiation to mature osteoblasts. The degree of methacrylation of GelMa together with the incorporation of copolymers VP/HEMA contributes to the cell development of pre-osteoblasts, with cytocompatibility levels equal to or higher than those of the reference materials, H-GelMa and L-GelMa homopolymers, while their properties may be tuned.

The results obtained in this work show the synergy between the different components of the ternary system proposed. This strategy has enabled us to uncover their potential as a new tunable and easily obtained biomaterial with the possibility of varying on demand their physicochemical properties while maintaining their cellular response.

## 4. Materials and Methods

This work was approached as a proof-of-concept by preparing new copolymers of GelMa with VP, HEMA and HEMA/VP comonomers, with two different concentrations (10 %wt. and 30 %wt.) and with 1:1 mixtures of HEMA/VP using the easy method of radical photopolymerization.

### 4.1. Materials and Reagents

All reagents and solvents were commercially available and used as received unless otherwise indicated. The following reagents and solvents were used:

For gelatin methacrylate synthesis and porous polymerized biomaterials; Gelatin from porcine skin (type A) (Sigma-Aldrich, Darmstadt, Germany, 99%), sodium carbonate (Sigma-Aldrich, 99%), sodium bicarbonate decahydrate (Sigma-Aldrich, 99%), sodium hydroxide (Sigma-Aldrich, 99%), methacrylic anhydride (MAA) (Sigma-Aldrich, ≥94%), milliQ water, deuterium oxide (D_2_O), dialysis tubing cellulose membrane (D9777-100FT, 14 KDa), 1-Vinyl-2-pyrrolidone (VP) (Sigma-Aldrich, ≥99%), 2-hydroxyethyl methacrylate (HEMA) (Sigma-Aldrich, ≥99%), Irgacure 2959 (PI) (Sigma-Aldrich, 98%). Gibco™ (Dublin, Ireland) Collagenase, Type I, powder (205 U/mg).

In relation with the cell line used, the mouse osteoblastic cell line (MC3T3-E1) was obtained from Sigma-Aldrich (Mouse C57BL/6 calvaria, Phenotype: Adherent, Karyotype: Not specified, Morphology: Fibroblast-like).

### 4.2. Synthesis of GelMa with Two Methacryloylation Degrees (DM)

The main purpose was to obtain two methacrylation degrees (lower—GelMa_LD_ and higher—GelMa_HD_, around 70% and 95%, respectively). For this, gelatin from porcine skin (type A) was dissolved at 10% (*w/v*) in a 0.2 M carbonate/bicarbonate buffer (CB) [31,32] buffer solution (pH 10) at 55 °C. After complete dissolution of the gelatin, MAA was added dropwise for 30 min to the protein solution under vigorous stirring (0.05 mL MMA/gr gelatin and 0.2 mL MMA/gr gelatin, for a lower and higher degree of methacrylation, respectively). The pH value was checked to confirm that it remained basic (pH 10). After 2 h of reaction, the solution pH was readjusted to pH 7.4 to stop the reaction, and the crude was dialyzed against milliQ water using a dialysis tubing cellulose membrane (with 14 KDa molecular weight cut-off) at 30 °C in milliQ water for 7 days. Subsequently, the dialyzed product was frozen and lyophilized to obtain GelMa with different degrees of methacryloilation (GelMa_HD_ and GelMa_LD_; Figure 1 and Table 1).

### 4.3. Degree of Substitution of GelMa Synthesized

With the aim of quantifying the DM of GelMa, ^1^H-NMR (400 MHz Varian) experiments were conducted. Around 50 mg of each lyophilized GelMa sample was dissolved in 1 mL of deuterium oxide (D_2_O) at 30 °C, together with gelatin from porcine skin.

For the quantification of DM, firstly the peak area of aromatic acids in the gelatin and GelMa samples were employed as a reference in each spectrum. Then, the peak area of lysine methylene protons which appear at around 2.9 ppm was used for calculation of the amount of methacryloyl groups (DM), using the following Equation (1) [9]:

(1) $$DM\left(\%\right)=\left(1-\frac{Area\;of\;lysine\;methylene\;of\;GelMa}{Area\;of\;lysine\;methylene\;of\;Gelatin}\right)\times 100$$

### 4.4. Foamed Biomaterial Preparation: GelMa and GelMa Copolymers

All materials used in this work were prepared conventionally by bulk radical polymerization, photochemically initiated, through self-crosslinking of GelMa monomer and copolymerization with 1-Vinyl-2-pyrrolidone (VP) and/or 2-hydroxyethyl methacrylate (HEMA) giving rise to the biomaterials whose constitution is depicted in Figure 3 and Table 1.

The general procedure to obtain hydrogels and final foamed materials was as follows:

A total of 0.5 gr of each GelMa (GelMa_LD_ and GelMa_HD_) were dissolved in 5 mL of milliQ water at 50 °C until GelMa was totally dissolved. Later, the required amount of each comonomer (VP and/or HEMA) was added to the GelMa solution, together with 1.5% weight of photoinitiator (I2959). The mixture was sonicated for 10 min to homogenize and degas the mixture. Finally, the solution was poured in circular Teflon molds and was irradiated with a UV lamp-curing system (365 nm) for 20 min. Solid membranes obtained were demolded and frozen. To obtain foamed biomaterials, membranes were lyophilized, and stored at 4 °C. In this way, the materials obtained are compiled in Table 1.

### 4.5. GelMa and GelMa Based Copolymers Characterization: Instrumentation and Methods

The degree of substitution in the synthesized GelMa monomers was performed through their *^1^H-NMR* spectra which were recorded on a Varian-Mercury 400 MHz using D_2_O as solvent.

Attenuated Total Reflectance/FT-Infrared Spectroscopy (ATR-FTIR) was used to characterize GelMa-based membranes of different copolymer compositions. For this purpose, ATR-FTIR spectra were registered using a Nicolet iS10 spectrophotometer (Waltham, MA, USA) outfitted with an ATR accessory (SMART Golden Gate attenuated total reflection)

Thermal properties of different GelMa biomaterials were analyzed by Thermogravimetric Analysis (TGA). TGA was performed in a TA Q-500 TA Instruments under nitrogen atmosphere, from 25 to 800 °C at a heating rate of 10 °C/min. The T_5_ (determined as the degradation temperature linked to a weight loss of 5%) and T_max_ degradation temperature were obtained.

To determine the presence of a macro-porous structure in foamed scaffolds, cross-sectional scanning electron microscopy (SEM) micrographs were undertaken in a JEOL 6400 Microscope-Oxford Pentafet super ATW system.

For the evaluation of mechanical properties, rheological measurements were performed using an Advance rheometer AR2000 with a 20 mm steel crosshatched plate. For this purpose, lyophilized and them swollen copolymers of VP/HEMA (10% and 30%) together with homopolymers H-GelMa and L-GelMa were analyzed. Two kinds of measurements were performed; firstly, strain sweep tests, in which the measures were taken between 0.1% and 100% strain and a constant frequency of 1 Hz, with the aim of determining the linear viscoelastic range (which is the range where the elastic and viscous moduli are independent of the strain). These tests were conducted at 25 °C.

Secondly, frequency sweep tests, with measurements that were performed in a frequency interval between 10^−2^–10^2^ Hz at a specific strain of 0.1% (within the linear viscoelastic range). In these tests, the elastic moduli (G′) was obtained.

### 4.6. Swelling Experiments

To investigate the water uptake of the GelMa scaffolds obtained, swelling experiments were performed. These were carried out at room temperature by dipping the different compositions described in Table 2 in deionized water, physiological phosphate buffer (PBS) and culture medium (DMEM). Samples were kept in each medium to reach the swelling equilibrium for at least 12 h and weighed (weight swollen). Afterwards, scaffolds were dried at 60 °C overnight and weighed again (weight dry).

The degree of swelling was calculated with the following Equation (2)

(2) $$\%\;swelling=\left(\frac{Weight\;swollen-Weight\;dry}{Weight\;dry}\right)\times 100$$

### 4.7. In Vitro Enzymatic Degradation

To observe the effect of in situ enzymatic degradation, firstly, materials were weighed dry after lyophilization (dry weight). Subsequently, they were allowed to swell in 2 mL of α-MEM medium supplemented with FBS and penicillin in an incubator at 37 °C overnight and weighed again (W_0_). Finally, materials were incubated in 2 mL of collagenase type I solution (0.3 mg/mL) at 37 °C. Samples were collected at 2 h, 4 h, 7 h, 24 h, 48 h, 72 h, 96 h and 120 h, and weighed to obtain the remaining wet weight (W_w_). In addition, after 24 h, samples were washed and lyophilized again to obtain the final dry weight.

The remaining weight was calculated by Equation (3)

(3) $$\%\;remaining\;weight=\left(\frac{W_{w}}{W_{0}}\right)\times 100$$

### 4.8. In Vitro Assays in Pre-Osteoblastic Mammalian Cells

All in vitro cell viability, proliferation and differentiation assays were performed with MC3T3-E1 mouse preosteoblastic cells (subclass 4, CRL-2593; ETCC, Manassas, VA, USA). For cell culture, α -Minimum Essential Medium (α-MEM, Sigma Chemical Company, St. Louis, MO, USA) supplemented with 10% fetal bovine serum (FBS, Gibco, Thermo Fisher Scientific, Wilmington, DE, USA), 1% penicillin-streptomycin and 5 mM L-glutamine (Gibco, Thermo Fisher Scientific, Wilmington, DE, USA) was used. Cells were maintained in a 5% CO_2_ atmosphere at 37 °C, washed with PBS, pH 7.4 (Gibco, Thermo Fisher Scientific, Wilming-ton, DE, USA) and trypsinized with 3 mL of 0.25% trypsin-EDTA (Gibco, Thermo Fisher Scientific, Wilmington, DE, USA). The resulting cell suspension was centrifuged for 10 min at 1200 rpm. Finally, the cells were resuspended with fresh medium of complete medium.

All experiments were performed in duplicate with three replicates per material and three cell controls. For this purpose, the materials were processed in disc shape (12 mm diameter and 1 mm height) by radical photopolymerization in Teflon molds (see Section 4.4) and cut into four equal parts. All materials were previously sterilized for half an hour on each side under UV radiation in a laminar flow cabinet.

After the sterilization process, the scaffolds were immersed for 1 h in culture medium to condition the materials and swollen in the oven at 37 °C and 5% CO_2_. The medium was then removed, and the swollen hydrogels were refilled with fresh complete medium. For cell viability, proliferation, and cytotoxicity assays, the preosteoblastic cell line MC3T3-E1 was seeded on the top surface of the swollen scaffold at the cell density corresponding to the assay. Incubation conditions for all assays were 37 °C with a 5% CO_2_ atmosphere.

### 4.9. In Vitro Cytocompatibility Assays in Pre-Osteoblastic Mammalian Cells

For this cellular test, preosteoblastic MC3T3-E1 cells were seeded in a 12-well plate onto swollen scaffolds at a density of 1 × 10^5^ cells/mL with a final volume of 2 mL of complete culture medium and maintaining them at 37 °C in 5% CO_2_ atmosphere. Cell cytocompatibility was evaluated by means of cell viability by Alamar Blue Method (AbD Serotec, Oxford, UK). Pre-osteoblastic cell growth onto the scaffolds was measured between 1and 7 days. With this aim, culture medium was removed, cells seeded were washed with PBS and exposed to 1:10 AlamarBlue^®^ (Invitrogen, Thermo Fisher Scientific, Wilmington, DE, USA) solution for 3 h in darkness at 37 °C and 5% CO_2_ before fluorescence measurements. Thus, the resulting product was collected and measured with a Synergy Multimode Plate Reader (BioTek Instruments, Winooski, VT, USA) with an excitation and emission wavelengths of 560 and 590 nm, respectively.

### 4.10. Morphological Studies by Confocal Laser Scanning Microscopy

Cell morphology and proliferation inside the porous structure of scaffolds was studied using fluorescence microscopy with a confocal scanning microscope OLYMPUS FV1200 (OLYMPUS, Tokyo, Japan) using a 60× FLUOR water dipping lens (NA = 1. For this purpose, MC3T3-E1 cells were seeded at a density of 1 × 10^5^ cells/mL over swollen scaffolds and incubated 24 h at 37 °C in a 5% CO_2_ atmosphere.

Procedure followed incubation, was to perform the removal of the media followed by washing twice with PBS. Cells then must be fixed using for this a solution of 4% paraformaldehyde in PBS for 30 min at 4 °C. Later, cells were washed with PBS and permeabilizated with 0.5% triton in water for 5 min at 4 °C. Then, the fixation medium was removed, and the cells were dyed with Phalloidin-ATTO 565 (dilution 1:40, Molecular Probes) for 10 min.

Next, the necessary volume of Fluoroshield^®^ with DAPI was taken to cover the scaffolds (1:1000, Sigma Aldrich, St. Louis, MO, USA), and was held for 10 min. Finally, each plate was washed twice and remained with PBS until analysis where DAPI and Atto 565-phalloidin dying were shown in blue and red, respectively. Micrographs were achieved using FV10-ASW (4.2 Version, Waltham, MA, USA) to develop a single 2D image, that can be converte into a TIF image file using multiple images obtained from each section in the Z axis and also using an algorithm which display the maximum value of pixels of each section in Z for each 1 m.

### 4.11. In Vitro Mineralization Assay

For matrix mineralization assay, the MC3T3-E1 osteoblastic cell line was seeded at a density of 1.5 × 10^4^ cells/mL and was cultured in 2 mL/well of differentiation culture medium: α-MEM (Sigma Chemical Company, St. Louis, MO, USA) supplemented with 10% fetal bovine serum (FBS, Gibco, Thermo Fisher Scientific, Wilmington, DE, USA), 1% penicillin-streptomycin, 5 mM L-glutamine (Gibco, Thermo Fisher Scientific, Wilmington, DE, USA), 50 µg/mL L-ascorbic acid (Sigma-Aldrich, St. Louis, MO, USA) and 10 mM β-glycerol-phosphate (Sigma-Aldrich, St. Louis, MO, USA). In this assay, the cells seeded on the scaffolds were cultured in differentiation medium for 11 days at 37 °C with a 5% CO_2_ atmosphere.

The detection of calcium deposits was performed in MC3T3-E1 pre-osteoblasts by Alizarin-Red staining. After 11 days of culture, cells were washed with PBS and then fixed with 75% ethanol for 1 h at 4 °C. Then, Cell cultures were dyed with a solution of alizarin red (40 mM) in distilled water (at pH 4.2) for 45 min at room temperature. Subsequently, to remove the excess of staining agent, cell monolayers were washed carefully with distilled water. Finally, the calcium deposits were dissolved using for that purpose a 10% solution of cetylpyridinum chloride in 10 mM sodium phosphate at pH 7.0. The resulting solution was collected and measured at 620 nm with a Synergy Multi-mode Plate Reader (BioTek Instruments, Winooski, VT, USA).

### 4.12. Statistical Analysis

The results were expressed as the mean SEM (standard error of the mean) of six specimens split in two independent experiments (three separate groups of samples in each one). Statistical analysis was performed with the nonparametric Kruskal–Wallis test and post-hoc Dunn’s test. For all statistical tests, a value of *p* < 0.05 was considered significant. Data were presented as mean ± standard deviation (SD). Error bars represented the SD of three independent samples.

## Figures and Tables

**Table 1:** Summary of GelMa-based polymers and copolymers obtained in this work by radical photopolymerization. Copolymer composition is given in % weight from GelMa monomer and expressed as follows: (%wt. GelMA/%wt. VP/%wt. HEMA/% Photoinitiator (PI)). For copolymers composed of VP and HEMA, the composition chosen was 1:1 VP/HEMA.

Foamed Biomaterials			
Higher DM		Lower DM	
Acronym	Composition	Acronym	Composition
H-GelMa	100/0/0/1.5	L-GelMa	100/0/0/1.5
H-GelMa-VP_10_	90/10/0/1.5	L-GelMa-VP_10_	90/10/0/1.5
H-GelMa-VP_30_	70/30/0/1.5	L-GelMa-VP_30_	70/30/0/1.5
H-GelMa-HEMA_10_	90/10/0/1.5	L-GelMa-HEMA_10_	90/10/0/1.5
H-GelMa-HEMA_30_	70/30/0/1.5	L-GelMa-HEMA_30_	70/30/0/1.5
H-GelMa-(VP/HEMA)_10_	90/5/5/1.5	L-GelMa-(VP/HEMA)_10_	90/5/5/1.5
H-GelMa-(VP/HEMA)_30_	70/15/15/1.5	L-GelMa-(VP/HEMA)_30_	70/15/15/1.5

**Table 2:** Thermogravimetric data for all biomaterials: The table below shows the temperature at 5% weight loss (T_5_) and the maximum decomposition temperature (T_max_) obtained from derivative weight loss curve (DTGA).

	T_10_ (°C)	T_max_ (1) (°C)	T_max_ (2) (°C)
H-GelMa	190	318	---
H-GelMa-VP_10_	244	325	427
H-GelMa-VP_30_	255	328	432
H-GelMa-HEMA_10_	252	325	411
H-GelMa-HEMA_30_	260	325	415
H-GelMa-(VP/HEMA)_10_	253	323	420
H-GelMa-(VP/HEMA)_30_	261	325	423
	T_10_ (°C)	T_max_ (1) (°C)	T_max_ (2) (°C)
L-GelMa	200	319	---
L-GelMa-VP_10_	248	324	430
L-GelMa-VP_30_	259	325	431
L-GelMa-HEMA_10_	249	324	413
L-GelMa-HEMA_30_	260	325	414
L-GelMa-(VP/HEMA)_10_	245	325	420
L-GelMa-(VP/HEMA)_30_	260	325	423

## Data Availability

Not applicable.
